# Influence of the distribution of fibrosis within an area of myocardial infarction on wave propagation in ventricular tissue

**DOI:** 10.1038/s41598-019-50478-5

**Published:** 2019-10-02

**Authors:** Cuiping Liang, Kuanquan Wang, Qince Li, Jieyun Bai, Henggui Zhang

**Affiliations:** 1 School of Computer Science and Technology, Harbin Institute of Technology, Harbin, Heilongjiang China; 2 School of Physics and Astronomy, The University of Manchester, Manchester, UK; 3 Space Institute of Southern China, Shenzhen, China; 4 Key Laboratory of Medical Electrophysiology, Ministry of Education, Collaborative Innovation Center for Prevention and Treatment of Cardiovascular Disease/Institute of Cardiovascular Research, Southwest Medical University, Luzhou, China

**Keywords:** Electrophysiology, Computational biophysics

## Abstract

The presence of fibrosis in heart tissue is strongly correlated with an incidence of arrhythmia, which is a leading cause of sudden cardiac death (SCD). However, it remains incompletely understood how different distributions, sizes and positions of fibrotic tissues contribute to arrhythmogenesis. In this study, we designed 4 different ventricular models mimicking wave propagation in cardiac tissues under normal, myocardial infarction (MI), MI with random fibrosis and MI with gradient fibrosis conditions. Simulation results of ideal square tissues indicate that vulnerable windows (VWs) of random and gradient fibrosis distributions are similar with low levels of fibrosis. However, with a high level of fibrosis, the VWs significantly increase in random fibrosis tissue but not in gradient fibrosis tissue. In addition, we systematically analyzed the effects of the size and position of fibrosis tissues on VWs. Simulation results show that it is more likely for a reentry wave to appear when the length of the infarcted area is greater than 25% of the perimeter of the ventricle, when the width is approximately half that of the ventricular wall, or when the infarcted area is attached to the inside or outside of the ventricular wall.

## Introduction

Serious cardiovascular disease (CVD) can induce SCD, causing more than 4 million deaths in Europe in 2016^1^. SCD is mainly induced by ventricular arrhythmia, especially among patients with MI^2,3^, in which the occurrence of atrial and ventricular tachyarrhythmias is strongly correlated with an increasing percentage of fibrosis in the heart tissue^2,4^. Additionally, during aging and under a variety of cardiac diseases, remodeling of cardiac structure could occur, resulting in a substantial increase in the proportion of extracellular matrix in the heart, a complex network including strands of fibrous collagen and elastin^5,6^. In the remodeling process, fibrosis is induced by the aggregation of fibroblasts, which occurs after MI^7,8^. As a result of fibrosis formation, the proportion of fibrotic tissue in the heart may increase up to 10 to 35%^9,10^. In fact, it has been found that the increase of fibrosis will give rise to a partial decoupling of muscle fibers, a zig-zag path of wave conduction and conduction slowing or blocking^11,12^, leading to arrhythmia. Furthermore, a clinical study has suggested that it is not only the amount but also the structure of the fibrosis that is an important factor for arrhythmogenesis^13^.

Several studies have investigated the influence of fibrosis on wave propagation in cardiac tissue. For example, ten Tusscher *et al*. showed that diffuse fibrosis slows wave propagation and increases tissue vulnerability to wave breaks and spiral wave formation^9^. Kazbanov *et al*. showed that a larger size and higher degree of heterogeneity of fibrosis are more likely to result in the formation of arrhythmias^14^. In addition, fibroblast density may be greater, especially in seriously injured areas such as infarcted or ischemic regions, and smaller in slightly injured areas of the tissue^15^, as has been found in rabbit ventricles^16^. Possible effects of a gradient array of fibrosis tissue on the formation and maintenance of arrhythmic reentrant waves in a 2D ideal normal ventricular tissue model were investigated by Zimik^15^. All of those simulations were based on a normal tissue model without considering the effect of MI. However, studies by Dongdong^2,17^, Arevalo^18^, Jalife^19^, and Connolly^12^ have shown that the electrophysiological properties in infarcted areas are different from those in normal areas. Many studies have investigated how the degree of heterogeneity in tissue with or without infarcted areas helps to induce arrhythmia^9,14,15,20,21^. However, to the best of our knowledge, no study has investigated the influence of different types of fibrosis distributions across the border of normal and infarcted tissue on arrhythmia. Moreover, how different distributions, sizes and positions of fibrosis tissues result in arrhythmic reentrant waves remain incompletely understood. Therefore, the present study designed a circular ring model to investigate the difference in wave propagation between random and gradient fibrosis distributions in MI ventricular tissues. In tissues with different fibrosis distributions, the degree of heterogeneity across the border of normal and MI areas is different and increases with the increase in percentage of fibrosis. For example, with the increase in the percentage of fibrosis, the degree of heterogeneity across the border of the circle further increases in random fibrosis tissue compared with that in gradient fibrosis tissue, resulting in larger changes in VWs in random fibrosis tissue in our following simulation. Furthermore, the study systematically investigated the influence of the size and the location of infarcted tissues on the VW, which is helpful for understanding the mechanism of arrhythmia in a MI patient. The role of infarcted tissues was verified in real 2D and 3D ventricular models with infarcted tissues.

## Results

### Structural design of gradient fibrosis distribution and circular ring tissue

Fibroblasts and myocytes couple in three different ways as proposed by Peter Kohl^22^, i.e., zero-sided coupling, single-sided coupling, and double-sided coupling^23–25^. In zero-sided coupling, myocytes and fibroblasts are not electrically coupled. In our simulation model, the MI area contains three cell types: normal, infarcted and fibrotic cells. Normal and infarcted cells have electrical properties, but excitation waves cannot propagate in the fibrotic tissue, which is similar to a zero-sided connection, where fibrotic cells in 2D cardiac tissue act as unexcitable obstacles represented by 1 × 1 grid points.

**Figure 1:**
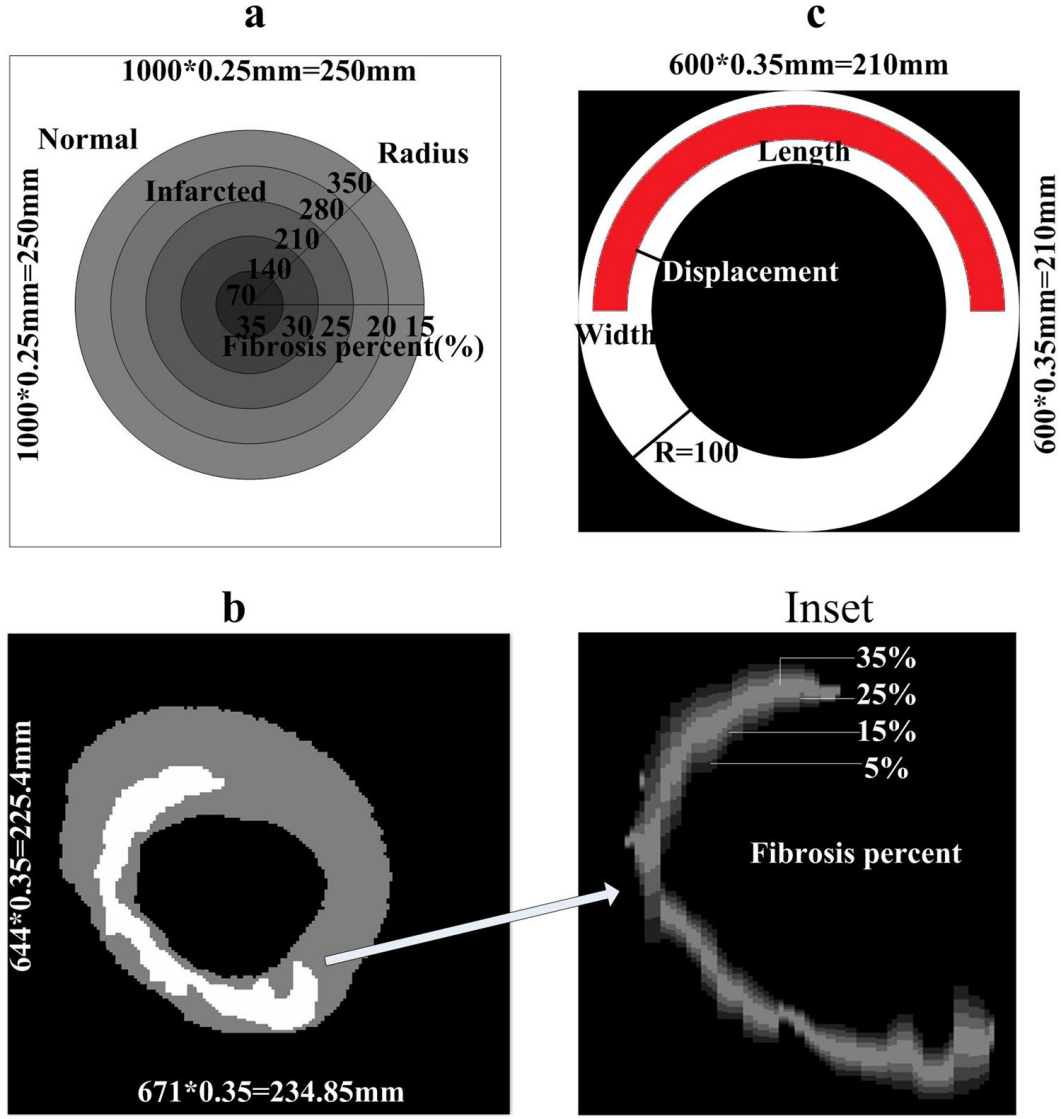
Gradient fibrosis distribution in 2D tissue and the structure of the infarcted area in the 2D circular ring model. (**a**) Structure of the 2D gradient fibrosis model in an ideal square tissue with a circular infarcted region. The infarcted region is separated into 5 layers; the percentage of fibrosis decreases outwards from the central region: 35%, 30%, 25%, 20% and 15%. (**b**) Structure of the infarcted region in a 2D, actual ventricular tissue. Inset shows the zoomed-in infarcted region from panel (b). The infarcted region is separated into 4 layers; the percentage of fibrosis decreases outwards from the central of the infarct: 35%, 25%, 15% and 5%. (**c**) Structure of the infarcted area in 2D circular ring tissue with different lengths (arc length), widths (width of ring strip) and locations of MI tissue (distance from the inside wall to the infarcted tissue).

To investigate the influence of increasing amounts of diffuse fibrotic tissue during the MI process, normal excitable cells were replaced by different percentages of fibrotic cells. In the 2D ideal tissue model (Fig. 1(a)), 5 circles with a radius from 70 to 350 pixels were designed at the center of the 2D ideal tissue. Within the 5 concentric circles, each with the same width, the fibrosis percentage decreased from 35% to 15% in steps of 5% from inside to outside. Thus, the average percentage of fibrosis within the entire circular area was approximately 21%. In a random fibrosis distribution, different percentages of fibrosis (6%, 11%, 16%, 23%, 26%, 28% and 31%) were selected to investigate the percentage of fibrosis on VWs. In the gradient fibrosis distribution, the fibrosis percentage in each ring was modified to make the average percentage of fibrosis within the circular area the same as that in the random fibrosis distribution (6%, 11%, 16%, 23% 26%, 28% and 31%). In real ventricular tissue, a gradient fibrous model was also designed to simulate wave propagation. In this case, the infarcted area was separated into four layers with 35%, 25%, 15% and 5% fibrosis from inside to outside, as shown in Fig. 1(b) and the Inset.

In Fig. 1(c), 2D circular ring tissues with different lengths, widths and locations of MI tissue were designed. The percentage of the width was computed as the width of the infarcted area divided by the radius of the ring tissue (as shown in Fig. 1(c), R). The percentage of the length was computed as the arc length of the infarcted area divided by the circumference of the circular ring. The position of the infarcted tissue was calculated as the distance to the inside wall of the ventricle divided by the radius of the ring tissue (as shown in Fig. 1(c), R).

### Differential wave propagation in 2D ideal random fibrosis and gradient fibrosis tissues

**Figure 2:**
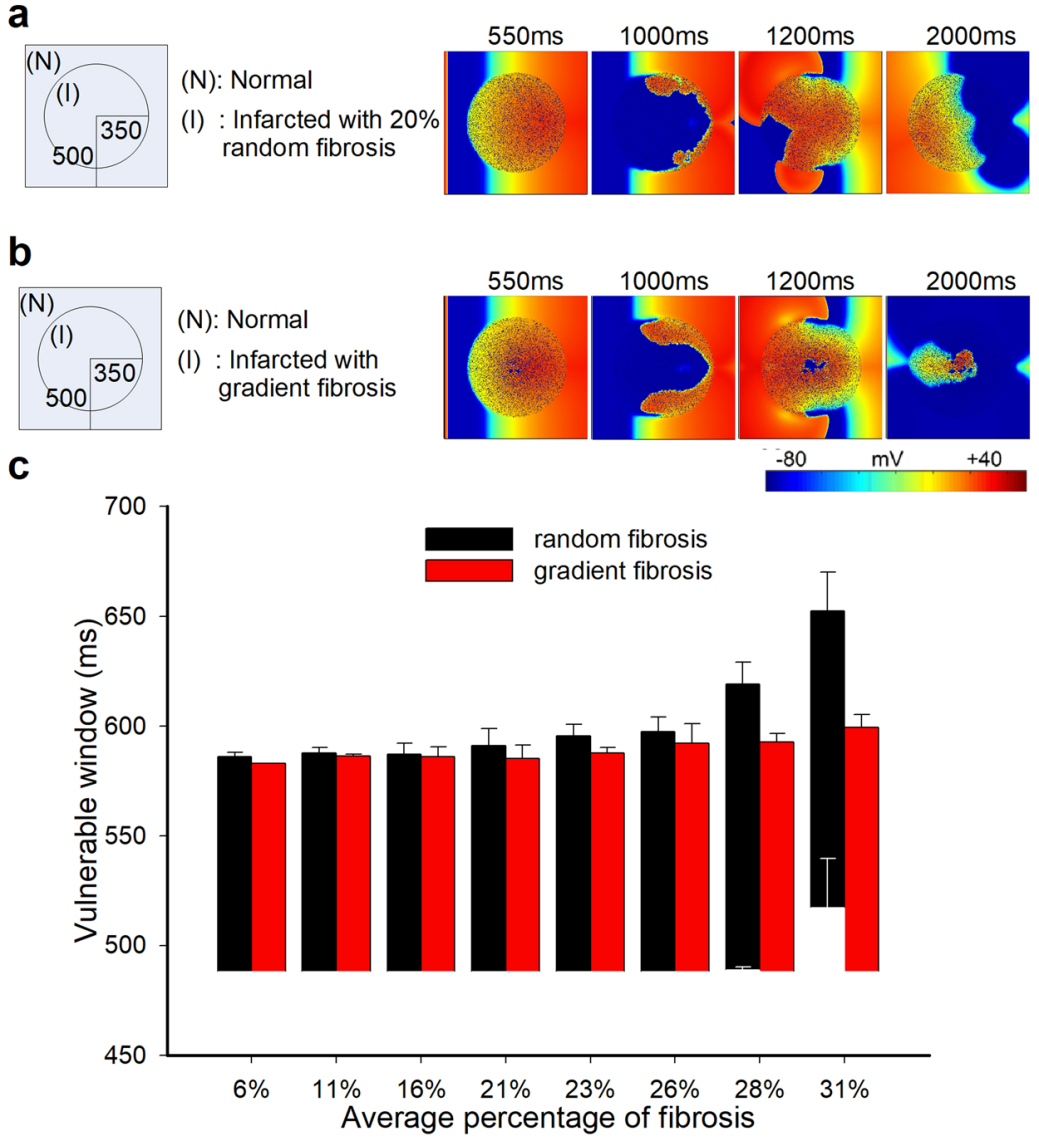
Simulation results in 2D ideal square tissue model. (**a**) Structural representation and wave conduction tested with two stimuli with S1-S2 intervals of 550 ms in the 2D ideal random fibrosis tissue at different times. (**b**) Structural representation and wave conduction tested with two stimuli with S1-S2 intervals of 550 ms in the 2D ideal gradient fibrosis tissue at different times. (**c**) Vulnerable windows of 2D ideal random and gradient fibrosis tissue ranging from 6% to 31% fibrosis.

**Figure 3:**
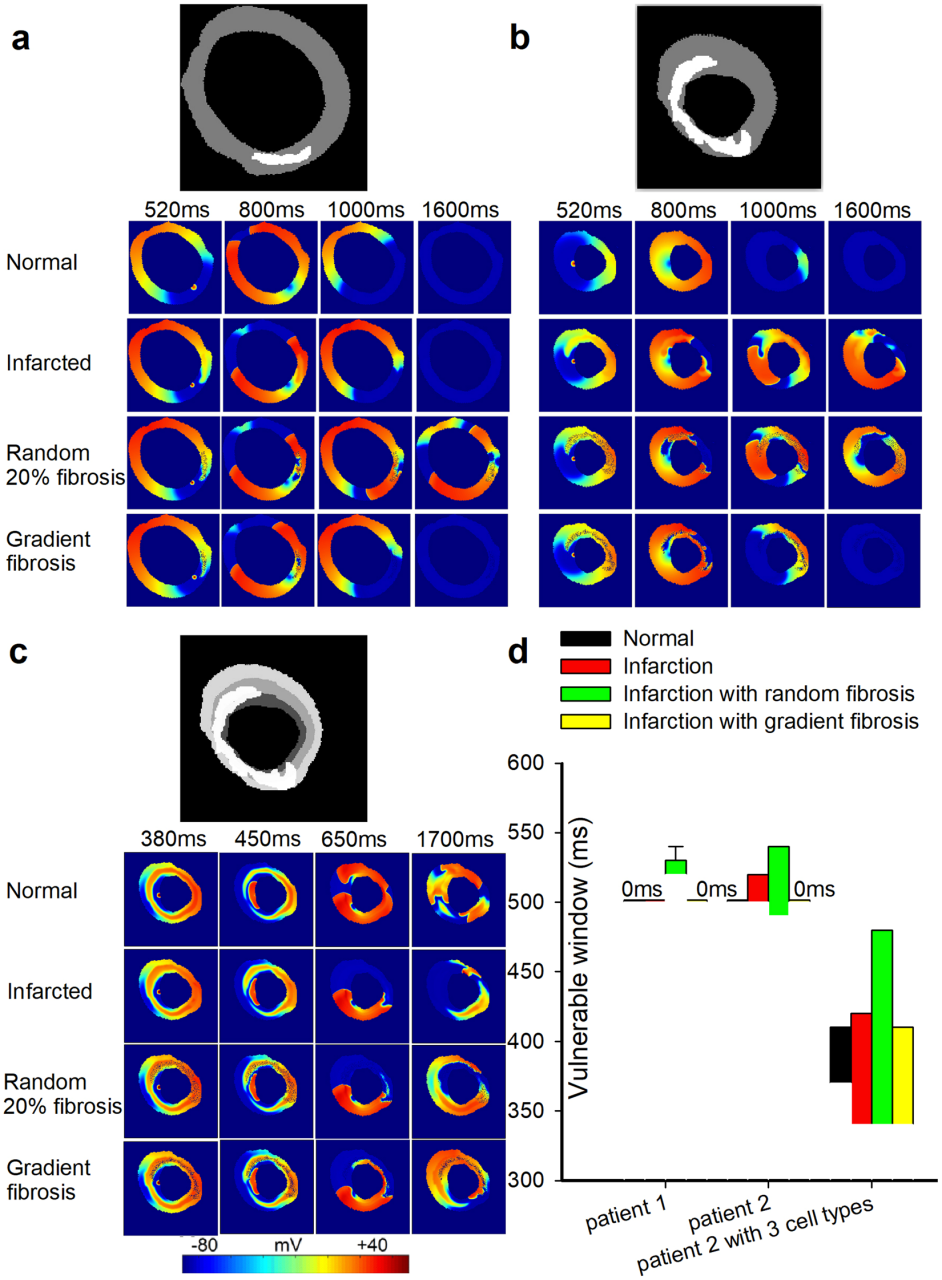
Wave propagation in the 2D actual ventricular tissue models under normal, infarcted, and MI with 20% random and gradient fibrosis conditions. (**a**) Structural representation and wave conduction tested with two stimuli with S1-S2 intervals of 500 ms under four conditions at different times in the tissue of patient 1, including only mid-myocardial cells. (**b**) Structural representation and wave conduction tested with two stimuli with S1-S2 intervals of 500 ms under four conditions at different times in the tissue of patient 2, including only mid-myocardial cells. (**c**) Structural representation and wave conduction under four conditions at different times in the tissue of patient 2, including three types of cells (endocardial cells, mid-myocardial cells and epicardial cells). (**d**) Vulnerable windows were calculated under four conditions in the tissue of patient 1 and patient 2 with only mid-myocardial cells as well as in the tissue of patient 2 with all three types of cells. A vulnerable window of 0 ms means that when the stimulus starts later than the lower bound of the S1-S2 interval, waves could conduct normally without the appearance of reentry waves. In the graph, the single line below the notation “0 ms” represents the lower bound of the S1-S2 interval where waves could conduct rather than a singular value of the vulnerable window. In addition, for conditions with no error bars shown, errors are too small to visualize. Both notations have the same meanings in the following figures.

**Figure 4:**
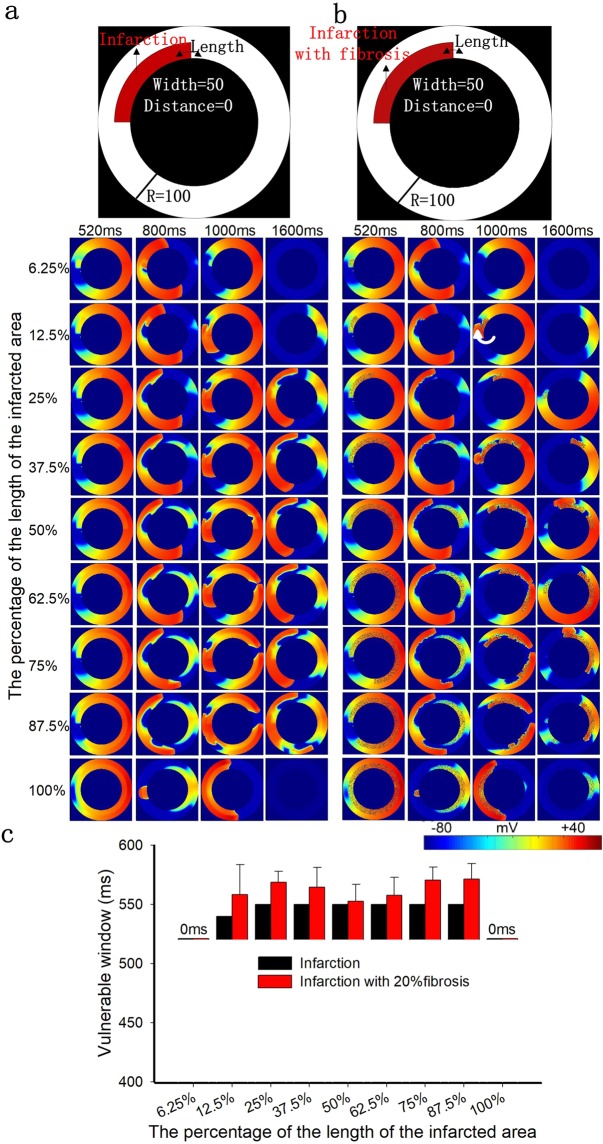
The role of the length of the infarcted area with a fixed width of 50% and a fixed position of 0% in excitation wave propagation in the circular ring tissue model. (**a**) Structural representation and wave conduction tested with two stimuli with S1-S2 intervals of 520 ms in the 2D ring model of infarcted tissue without fibrosis at different times. (**b**) Structural representation and wave conduction tested with two stimuli with S1-S2 intervals of 520 ms in the 2D ring model of infarcted tissue with 20% fibrosis at different times. (**c**) Change in the size of the vulnerable windows in the circular ring tissue with and without 20% fibrosis as a function of the length of the infarcted tissue. Black bars: infarcted tissue without fibrosis; Red bars: infarcted tissue with 20% random fibrosis.

**Figure 5:**
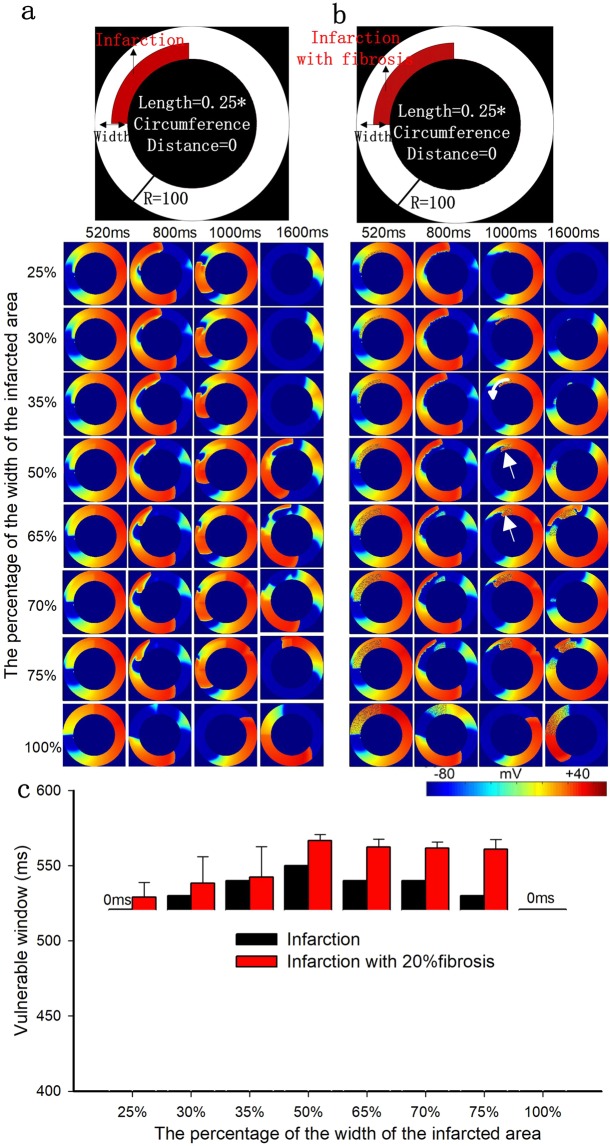
The role of the width of the infarcted area with a fixed length of 25% and a fixed position of 0% in excitation wave propagation in the circular ring tissue. (**a**) Structural representation and wave conduction tested with two stimuli with S1-S2 intervals of 520 ms in the 2D ring model of infarcted tissue without fibrosis at different times. (**b**) Structural representation and wave conduction tested with two stimuli with S1-S2 intervals of 520 ms in the 2D ring model of infarcted tissue with 20% fibrosis at different times. (**c**) Change in the size of the vulnerable windows in the circular ring tissue with and without 20% fibrosis as a function of the width of the infarcted tissue. Black bars: infarcted tissue without fibrosis; Red bars: infarcted tissue with 20% random fibrosis.

**Figure 6:**
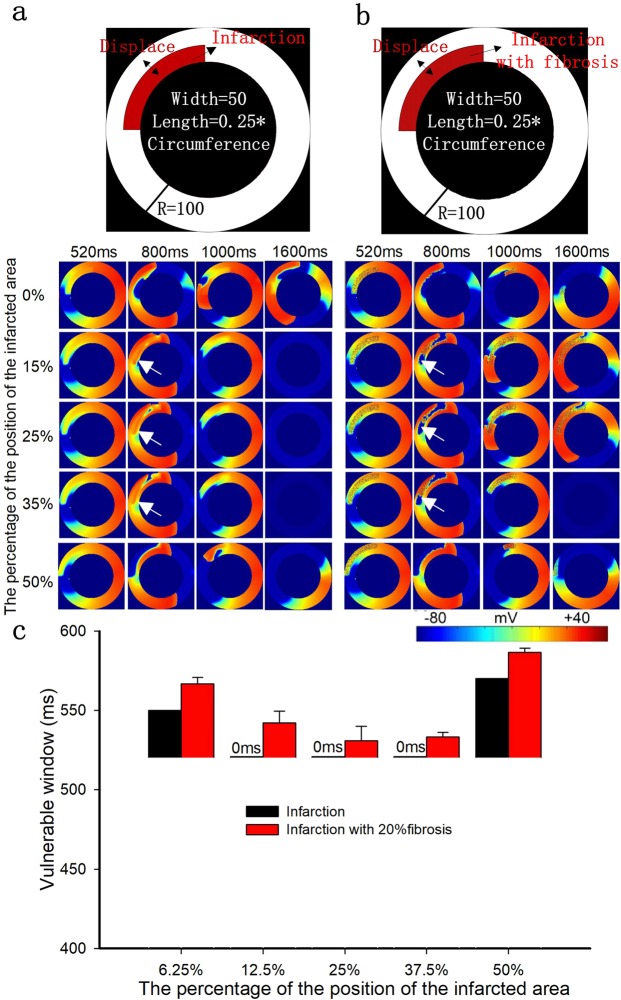
The role of the position of the infarcted area with a fixed length of 25% and a fixed width of 50% in excitation wave propagation in the circular ring tissue. (**a**) Structural representation and wave conduction tested with two stimuli with S1-S2 intervals of 520 ms in the 2D ring model of infarcted tissue without fibrosis at different times. (**b**) Structural representation and wave conduction tested with two stimuli with S1-S2 intervals of 520 ms in the 2D ring model of infarcted tissue with 20% fibrosis at different times. (**c**) Change in the size of the vulnerable windows in the circular ring tissue with and without 20% fibrosis as a function of the position of the infarcted tissue. Black bars: infarcted tissue without fibrosis; Red bars: infarcted tissue with 20% random fibrosis.

**Figure 7:**
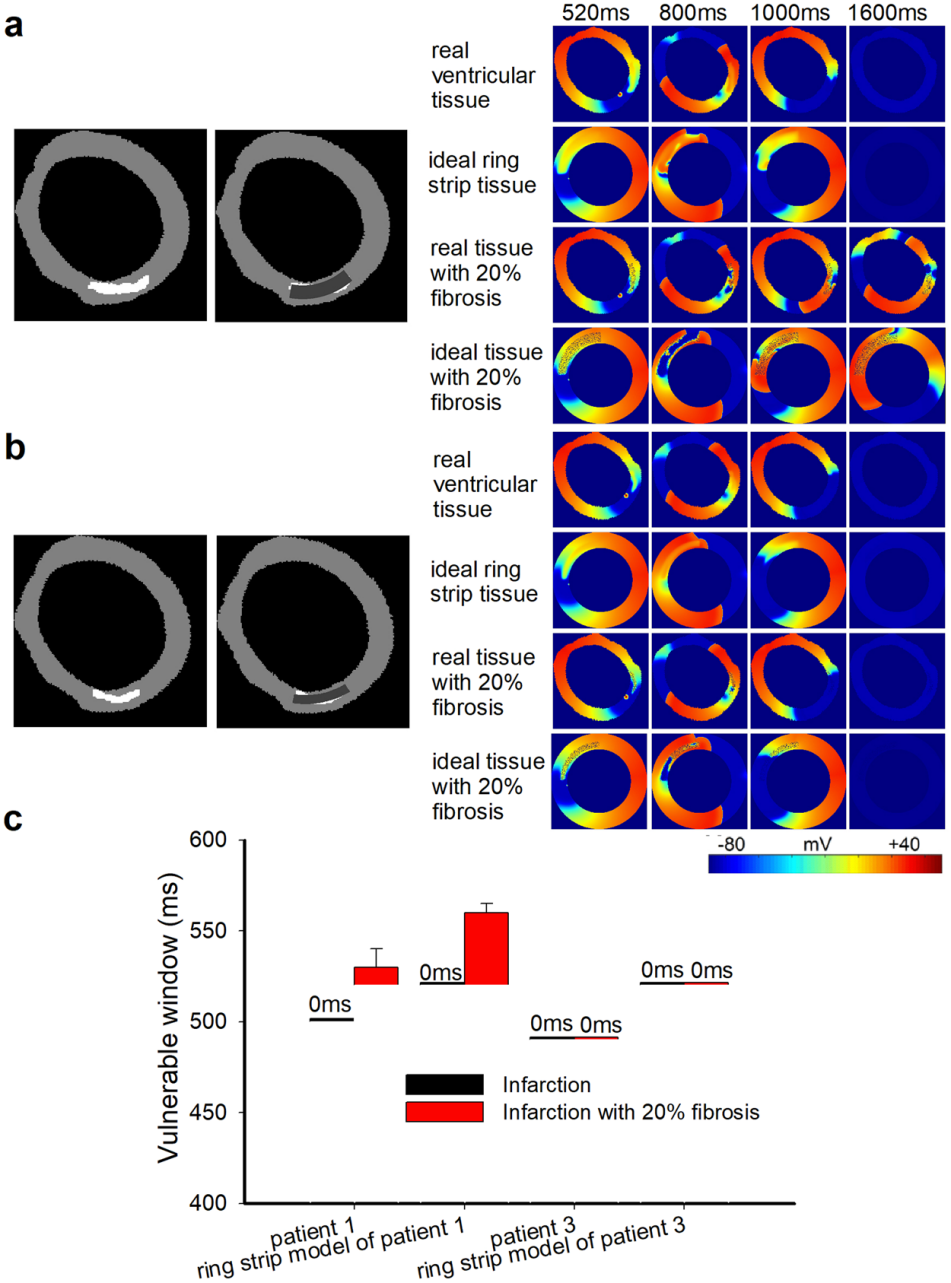
Wave propagation in the 2D, actual ventricular tissue and ideal circular ring tissue models. (**a**) Structural representation and wave propagation tested with two stimuli with S1-S2 intervals of 520 ms in the 2D actual ventricular tissue model of patient 1 and in the corresponding ideal ring strip tissue model with and without 20% fibrosis. (**b**) Structural representation and wave propagation tested with two stimuli with S1-S2 intervals of 520 ms in the 2D actual ventricular tissue model of patient 3 and in the corresponding ideal ring strip tissue model with and without 20% fibrosis. (**c**) The vulnerable windows of the actual infarcted tissues of patient 1 and patient 3 and their corresponding ring strip tissues with and without 20% fibrosis. Black bars: tissue without fibrosis; Red bars: tissue with 20% random fibrosis.

In 2D tissues, reentrant waves were induced by applying two stimuli at the same position. The vulnerable period (or window) was calculated as the coupling time interval between the two stimuli (i.e., the time interval between S1 and S2), during which a reentry wave can be induced by the S2 stimulus^26–30^. In our manuscript, excitation waves that were sustained more than 800 ms were defined as reentry waves. Starting from 480 ms, the VW was tested by increasing the stimulus interval by 1 ms each time, as shown in Fig. 2 (5 ms each time in Figs 3–7), using 6 different random seeds in Figs 2–7, except for Fig. 4 (11 random seeds). We investigated the effect of different fibrosis distributions on the genesis of reentry waves. Reentry waves appeared in both random fibrosis and gradient fibrosis tissues, as shown in Fig. 2(a,b). VWs increased slowly in both cases, with the percentage of fibrosis ranging from 6% to 31%. At low levels of fibrosis, the VWs between two different fibrosis distributions were similar, as shown in Fig. 2(c). However, the VWs significantly increased in random fibrosis tissues at high levels of fibrosis but did not increase in gradient fibrosis tissues. Furthermore, the waves were blocked at the center of the tissue with gradient fibrosis ranging from 23% to 31% due to the high fibrosis percentage. Overall, the risk of arrhythmia in the tissue with random fibrosis was higher than that in the tissue with gradient fibrosis at high levels of fibrosis.

### Differential wave propagation in 2D real ventricular tissues under normal, infarcted, random fibrosis and gradient fibrosis conditions

To test our hypothesis that the risk of arrhythmia in tissue with random fibrosis is higher than that in tissue with gradient fibrosis, further simulations were carried out on real ventricular tissues. Figure 3(a,b) show wave propagation in the ventricular tissues of patients 1 and 2, respectively, under normal, infarcted, and infarcted with 20% random fibrosis or 20% gradient fibrosis conditions. The simulation results showed that the VWs of the normal and gradient fibrosis ventricular tissues of patients 1 and 2 were both 0 ms, while the VWs of the ventricular tissue with 20% random fibrosis were the largest in both patients 1 and 2, with durations of 10 ms and 50 ms, respectively. The VWs of the MI tissue ranged from 500 ms to 520 ms in patient 2. The VW of the MI tissue was wider than that of the normal tissue because of the higher heterogeneity in the MI tissue. Furthermore, the VW in the MI tissue with fibrosis was larger compared with the MI tissue without fibrosis. Figure 3(c) shows simulations of ventricular tissue from patient 2 with three cell types (endocardial cells, middle cells and epicardial cells, with a percentage ratio of 25:35:40 from inside to outside^31^). Similar results were observed with the three cell types, where the maximum value of the VW appeared in the ventricular tissue with 20% random fibrosis but with a much longer duration of 140 ms compared with the case with only a single cell type. In normal tissue and infarcted tissue without and with gradient fibrosis, the VWs were 40 ms, 80 ms and 70 ms, respectively, as shown in Fig. 3(d).

These results are consistent with our conclusion that the VW in tissue with random fibrosis is higher than that in tissue with gradient fibrosis. This can be explained by the fact that although the heterogeneity was higher within infarcted tissue in gradient fibrosis compared with random fibrosis, the heterogeneity was lower across the border between normal and infarcted tissue in gradient fibrosis, leading to a smaller VW. Moreover, an increase in electrical heterogeneity under the condition including three types of cells, further enlarged VWs under every condition, as shown in Fig. 3(c,d), but this did not change our conclusion. In addition, the VW of patient 2 was wider than that of patient 1, which may be attributed to the different sizes and/or shapes of the infarcted tissue. However, it is difficult to determine how the shape of the infarcted area influenced the VW. Therefore, in the following sections, we simulated how different sizes and positions of the infarcted area influence the VW.

### Roles of different sizes and positions of infarcted areas on excitation wave propagation in the circular ring tissue

As the size and position of the infarcted area are very important factors in the formation of reentry waves, we designed 2D circular ring tissues with different lengths, widths and locations of the MI to simulate the influence of these factors on wave propagation.

First, we investigated how the length of the infarcted area influenced wave propagation. Since the conduction velocity in the infarcted tissue is slower than that in the normal tissue (as shown in Supplementary Fig. S1(d)), the infarcted tissue provides a potential structure for a reentry wave, as shown in Fig. 4(a). In addition, the conduction velocity in the infarcted tissue with fibrosis is lower than that in the fibrosis-free infarcted tissue (as shown in Supplementary Fig. S1(d)), resulting in slower wave propagation in the infarcted tissue with fibrosis, as shown in Fig. 4(b). The simulation results showed that the changes of the VW in tissues with and without fibrosis were similar (increased at the beginning and then reached a constant value) as the length of the infarcted tissue increased. Figure 4(c) shows that the VW in the nonfibrotic infarcted tissue was constant over lengths of the infarcted area ranging from approximately 25% to 90% of the circumference of the circular ring tissue. And, in the infarcted tissue with 20% random fibrosis, the VW was also nearly constant over lengths of the infarcted area ranging from approximately 10% to 90% of the circumference of the circular ring tissue.

In infarcted tissues with and without fibrosis, the trends of VW variation were similar with increasing width of the infarcted area (as shown in Fig. 5). The VW first increased and then decreased gradually after reaching a width of 50%. At 50%, the normal and infarcted areas were balanced, and the heterogeneity along the radial direction was the largest (as shown in Fig. 5(a,b)). In this condition, the size of the resting tissue that allowed a backward excitation wave induced by slow repolarization within the infarcted area reached a maximum (as shown by the arrow in Fig. 5(b), panel 50%, 1000 ms). The heterogeneity along the radial direction gradually decreased when the width was greater or less than 50%. In infarcted tissue with 20% fibrosis, due to the further delay of wave propagation, the VW was much larger than that in fibrosis-free MI tissue, especially when the width of the infarcted tissue was large. When the widths of infarcted areas were small (25%) or large (100%), the heterogeneity along the radial direction was not large enough to produce reentry (as shown in Fig. 5(c)).

Finally, Fig. 6(a,b) show how the position of the infarcted area (i.e., the percentage of distance from the inside wall to the radius of the circular ring) influenced wave propagation. It showed that when the infarcted tissue was attached to the outside of the ventricular wall (a distance of 50%), the VW reached its maximum value in infarcted tissue with or without fibrosis. The second largest VW appeared when the distance was 0% (i.e., attached to the inside wall). The VW was small and nearly constant between percentage distances of 15% and 35% in infarcted tissue with and without fibrosis (as shown in Fig. 6(c)). In this range, the infarcted areas were in the middle of the circular ring, with normal tissue located at both sides, providing two normal-tissue pathways for wave propagation (as shown by arrows in Fig. 6(a), panel 15–35%, 800 ms). Thus, in this range, there were no reentry waves observed in the nonfibrotic infarcted tissue. However, in infarcted tissue with 20% random fibrosis, the repolarization is slower than in the nonfibrotic infarcted tissue, creating an opportunity to induce reentry waves (as shown by arrows in Fig. 6(b), panel 15–35%, 800 ms).

### The influence of the infarcted area on wave propagation in the 2D and 3D ventricular models

To test our previous hypothesis, we designed a regularly shaped infarction structure (arc-shaped) similar to a real scar in ideal ring tissue and compared wave propagation in the real infarcted tissue from patients with that in ideal ring tissue. In patient 1, the wave propagation in the actual scar tissue was compared with that in an ideal ring tissue with an infarcted area having length, width and distance percentages of 25%, 60% and 10%, respectively, as shown in Fig. 7(a). The VWs in both the ideal and real infarcted ventricular tissue without fibrosis were 0 ms (as shown in Fig. 7(c)). In real infarcted tissue with 20% fibrosis, the width of the VW was 10 ms (from 520 ms to 530 ms). In ideal infarcted tissue with 20% fibrosis, the VW ranged from 520 ms to 560 ms. Similarly, in patient 3, the ideal simulation was based on a circular ring with length, width and distance percentages of 25%, 35% and 10%, respectively, as shown in Fig. 7(b). No reentry waves were observed, and the VWs in the infarcted tissue with or without fibrosis were both 0 ms (as shown in Fig. 7(c)), as the infarcted tissue was too thin, which is consistent with our previous simulation. The simulation results suggested that only infarcted areas with a sufficient length, width and distance could allow a reentry wave to appear.

**Figure 8:**
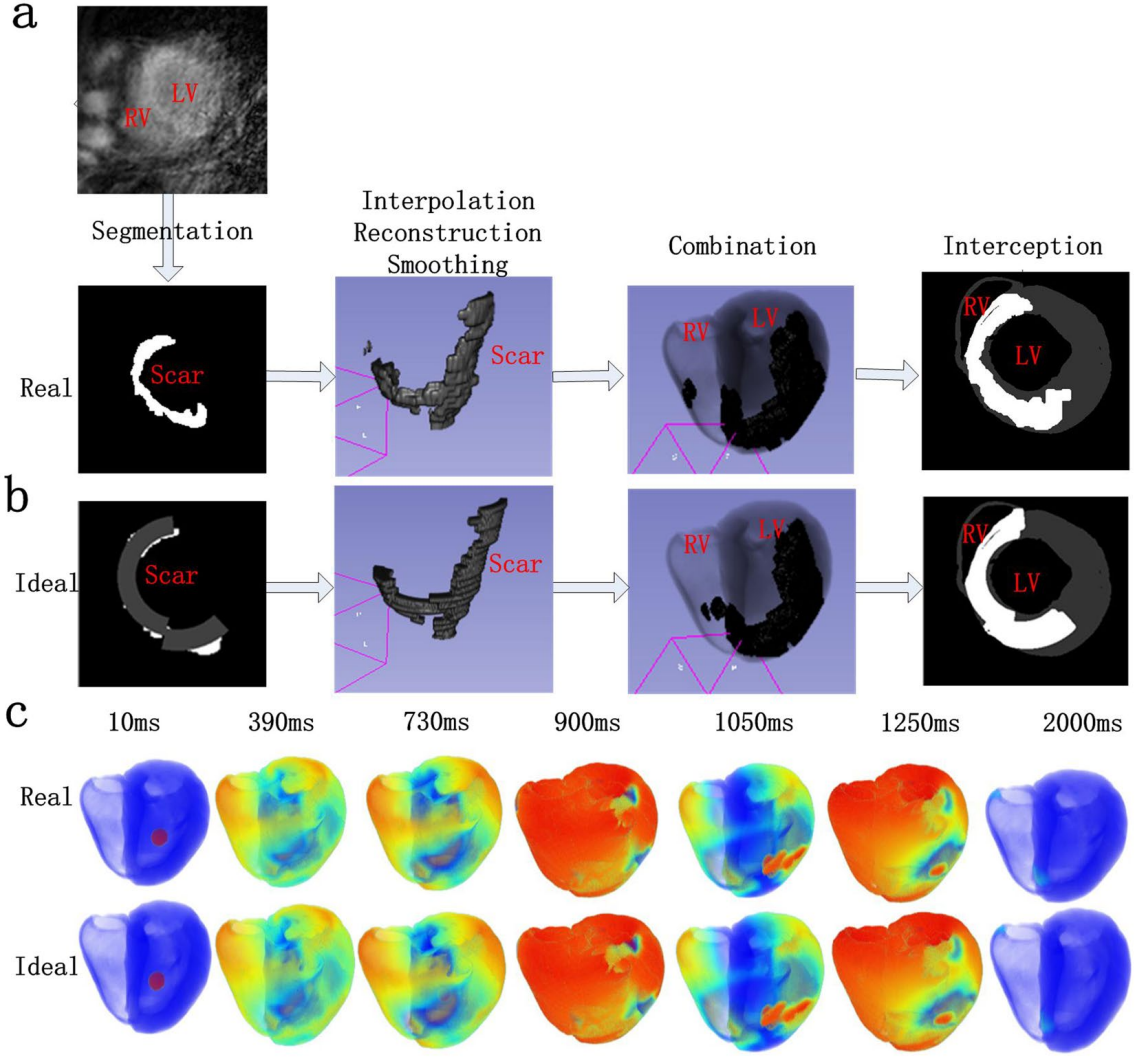
Modeling process and wave propagation in 3D real ventricular models. (**a**) Real scar modeling process in 3D real ventricular models, including segmentation, interpolation, reconstruction, smoothing and combination. (**b**) Ideal scar modeling process in 3D real ventricular models, including 2D graphic plotting, interpolation, reconstruction, smoothing and combination. (**c**) Wave propagation in 3D real ventricular models with real and ideal 20% fibrotic scar areas tested with three stimuli at 0 ms, 387 ms and 720 ms in turn at the same position (LV: left ventricle; RV: right ventricle).

We also tested our hypothesis in a 3D human ventricular model. First, real 2D scar geometry was segmented, interpolated, reconstructed and smoothed to form real 3D scar tissues, as shown in Fig. 8(a). Second, we designed an ideal shape for the infarction structure (arc-shaped) similar to a real scar in ideal 2D scar tissue and constructed ideal 3D scar tissues using the same steps (interpolation, reconstruction and smoothness), as shown in Fig. 8(b). The real and ideal 3D scar tissues were incorporated into a 3D human ventricular model as shown in Fig. 8(c) to investigate the role of infarcted tissue on wave propagation in the 3D heart. Excitation waves were initiated by employing stimuli at the intramyocardial region in the 3D human ventricular model. As shown in Fig. 8(c), reentry waves were observed in both the real and ideal 3D human ventricular models, which is consistent with our previous simulation. Reentry waves occurred at 1050 ms and disappeared at 2000 ms, as shown in Fig. 8(c). The patterns of wave propagation in both real and ideal scar models were similar.

## Discussion

Fibrosis may slow or block wave conduction in cardiac tissue, which likely induces arrhythmias^6,32–34^. Additionally, the fibrosis distribution, in addition to the average level of fibrosis, is also an important factor inducing arrhythmias^9,19^. In this work, we investigated how different heterogeneous distributions (random or gradient) of fibrotic tissue influence the genesis of arrhythmic reentry waves. In addition, this study showed in detail how the size and position of an infarcted area influence the occurrence and propagation of reentry waves, which is important for understanding the mechanism of fibrosis-induced arrhythmia in a MI patient.

Most previous studies were based on random fibrosis distributions; however, the distribution of fibrosis in actual tissue may not be random^15,16^. In our study, the effect of omnidirectional gradient fibrosis, which may be a more realistic realization of gradient fibrosis structure^16^, was mimicked and compared with random fibrosis.

Kazbanov *et al*.^14^ ran simulations that showed that a larger size and a larger degree of heterogeneity made the formation of arrhythmias more probable. In our simulations of 2D ring tissue, VWs increased slowly in both random and gradient fibrosis tissue when heterogeneity increased, which is consistent with the results of Kazbanov. Our results also show that differences in the VWs between two different distributions of fibrosis are small at low levels of fibrosis, as shown in Fig. 2(c). However, the VW significantly increased in random fibrotic tissue at a high level of fibrosis, but not in gradient fibrotic tissue. The random fibrosis induced electrical heterogeneity across the border between the normal and infarcted tissue, while the electrical heterogeneity within the infarcted region was relatively small. However, in the gradient fibrosis distribution, the electrical heterogeneity within the infarcted region was larger than that in the random distribution, while the electrical heterogeneity across the border between the normal and infarcted tissue was relatively smaller than that in the random distribution. The simulation results suggest that the risk of arrhythmia depends more on the acute change of heterogeneity across the border between different tissues than on the heterogeneity within the infarcted area.

We further verified our conclusion in real 2D ventricular tissues. First, it was shown that the VW was widest in MI tissue with 20% random fibrosis rather than in tissue with gradient fibrosis, as shown in Fig. 3(d). The VWs of gradient fibrosis in patient 1 and patient 2 were both 0 ms. In addition, in our simulation of real ventricular tissue using three cell types, the simulation results were similar to that in real ventricular tissue only with mid-myocardial myocytes, as shown in Fig. 3(c,d). In this condition, the maximum value of the VW (140 ms) appeared in tissue with 20% random fibrosis, and the VW in the infarcted tissue with gradient fibrosis was 70 ms, as shown in Fig. 3(d). Although the maximum fibrosis amplitude was larger and the spatial heterogeneity was higher in the MI region with gradient fibrosis, the VW was smaller compared with that in the tissue with 20% random fibrosis. The simulation results in the real ventricular tissues were consistent with our conclusion from the ideal 2D tissue. In this case, especially at high levels of fibrosis, a gradient distribution has a more protective effect compared with a random distribution.

In addition to the different distributions, it has been demonstrated that the size and position of the infarcted areas may have significant influences on the occurrence of reentry waves. Therefore, this study designed 2D, ideal circular ring tissue with MI regions of different lengths, widths and locations to simulate how these factors influence wave propagation.

There was a gradual increase in the VW and then reached a constant value in both cases (infraction and infarction with 20% fibrosis) (as shown in Fig. 4(c)) as the length of the infarcted area increased. Reentry waves were mainly created in the upper left region of the circle (as shown by the arrow in Fig. 4(b), panel 12.5%, 1000 ms), close to the border of normal and infarcted tissue where the conduction velocities (CVs) are significantly different between the two tissues. Therefore, once reaching a threshold, the VW kept a constant with further increase of the length of infarcted tissues. Since the CVs in infraction tissues with and without 20% fibrosis were different, the slope and point from where the VW became a constant would then be different (25% and 12.5% for tissues of infraction and infarction with 20% fibrosis, respectively). Furthermore, the VW of infarction tissue with 20% fibrosis case would be larger due to the zig-zag wave conduction and conduction slowing, leading to a wider spectrum that can cause break-up and arrhythmia.

The trends of the VWs in infarcted tissue with and without fibrosis were similar when the width of the infarcted area was changed. The major difference is that the conduction velocity is slower in the infarcted tissue with fibrosis, leading to larger VWs. As the width of the infarcted area increased from 25% to 50%, it takes longer for waves arriving at the infarcted area to propagate to the end of the infarcted area (as shown by the arrow in Fig. 5(b), panel 35%, 1000 ms). Therefore, the VW increased with increasing infarct width in this range. At a width of 50%, the VW reached its maximum value in the infarcted tissue with 20% fibrosis. As the width increased further, a slow repolarization in the infarcted area suppressed the backpropagation of waves (as shown in Fig. 5(b), panel 65%, 800 ms), and reentry waves occurred at the end of the infarcted area (as shown by the arrow in Fig. 5(b), panel 65%, 1000 ms). Consequently, the VW decreased with increasing infarct width from 50% to 100%. Therefore, the vulnerability inducing reentry waves is not directly proportional to the width of the infarcted area but is instead the result of the dynamic balance between the width and the consequent slow repolarization. When normal tissue and infarcted tissue both take up half of the width, the relative heterogeneity in the radial direction is the largest. Therefore, the probability of inducing reentry waves is at its maximum.

The results for the position of the infarcted area were similar for the infarcted tissue with and without fibrosis. In the fibrosis-free infarcted tissue with distances ranging from 15% to 35%, when the waves backpropagated to the end of the infarcted area, the infarct did not repolarize completely, and so these waves could not induce reactivation of the normal tissue again (as shown by arrows in Fig. 6(a), panel 15–35%, 800 ms). However, the slower repolarization in the infarcted tissue with fibrosis compared with that in the fibrosis-free infarcted tissue created an opportunity to induce reentry waves (as shown by arrows in Fig. 6(b), panel 15–35%, 800 ms). However, most of the up and down waves counteracted each other at the end of the infarcted area in the forward direction, and so there were few waves that propagated to the infarcted area. This is the reason why the VW in the infarcted fibrotic tissue at the abovementioned distances is slightly small. When the infarcted tissues with and without fibrosis were attached to the inside or outside of the myocardial tissue (as shown in Fig. 6(c)), the VW reached its maximum value. This is because the excitation waves from the normal tissue could propagate to the infarcted areas successfully and induce reentry waves.

In conclusion, it is more likely for a reentry wave to appear when the length, width and position of the infarcted tissue are in regions that increase the spatial heterogeneity in the corresponding direction. In addition, the VWs increased more in fibrotic tissue than in fibrosis-free MI tissue due to the further increased heterogeneity induced by fibrosis. These results were verified in 2D and 3D real ventricular models with infarcted areas.

Many previous studies have focused on the role of fibrosis in arrhythmia. For example, the work of Gabriel Balaban^35^ developed a computational model by incorporating random microstructures of fibrotic scarring in non-ischemic dilated cardiomyopathy (NIDCM). Their study highlighted the role of fibrosis type (interstitial or replacement fibrosis) and density and the slowing of local conduction in reentry induction in NIDCM. Although their work was based on 2D real ventricular tissues, the role of different heterogeneous distributions (random or gradient) of fibrosis tissue and of the size and position of infarcted tissue in the occurrence and propagation of reentry waves were not discussed. In this study, we provided a more systematic theory for understanding the mechanism of arrhythmia. Therefore, their research and our study are complementary.

An important limitation of our simulations is that we considered only the condition in which myocytes and fibroblasts were not electrically coupled. For a better description of physiological status, the potential of fibroblasts and how they couple electrically with myocytes need to be considered in future simulation. Furthermore, in our simulation of circular ring tissue, the shape of the infarcted area was designed as a ring strip, and ventricular tissue was designed as a circular ring, which are both more or less ideal. Moreover, the number of samples in our simulation obtained from patients was small, and more data should be used to verify our work. However, the limitations listed above do not alter the conclusions of this study.

## Methods

### Electrophysiological model in single cell simulation

The TP06 model was used in our simulation, which was based on human experimental data^36,37^. According to experimental recordings^2,20^ which are based on clinical trial data^38–40^, modifications to the ionic model of the infarcted area were implemented. Peak sodium current (I_Na_), peak L-type calcium current (I_CaL_), and peak potassium currents (I_Kr_ and I_Ks_) were decreased to 38%, 31%, 30% and 20% of the original values in the Ten Tusscher model, respectively. In single cell simulations, pacemaking APs were elicited by pre-pacing the models one hundred times to reach a stable steady state. And 100 stimuli were applied for a pacing cycle length of 1000 ms. Here, we simulated the AP of middle-cells in normal and infarcted cell models.

### Mathematical models

By using parabolic partial differential equation (PDE), the cell models were coupled into a mono-domain model with isotropic diffusion of cardiac electrophysiology to describe the reaction-diffusion property of the cardiac dynamics^41^. The main equation is:

1 $$\frac{\partial {V}_{m}}{\partial t}=\nabla \cdot D\nabla {V}_{m}-\frac{{I}_{ion}}{{{\rm{C}}}_{m}}$$

where *C*_*m*_ = 1 *μF*/*cm*^2^ is the capacitance, D is the effective diffusion constant, and the value of D is set to be 0.54 *mm*^2^/*ms*, *I*_*ion*_ is the total transmembrane current^36,37^.

### Numerical methods

To solve the PDE in equation 1, the forward Euler method was used with a time step (Δt) of 0.02 ms and different space steps (Δx = Δy). The spatial resolutions of the different 2D tissue structures were as follows: 2D ideal tissue model: Δ*x* = Δ*y* = 0.25 mm; and 2D circle ring and ventricular model: Δ*x* = Δ*y* = 0.35 mm. The spatial resolution in the 3D models was Δ*x* = Δ*y* = 0.33 mm. In both models, the diffusion coefficient (D; the determinant of conduction velocity) was 0.154 mm^2^/ms; Neumann boundary conditions (i.e., no-flux) and the stability criterion (e.g., DΔt/Δx^2^ < 1/2d, where d is the dimensionality of the simulation) were used^41^ in our experiments. Simulations were carried out on an Intel core i703930K 64-bit CPU system with 64 GB memory. GPU acceleration was applied to solve the parallel equation more efficiently^42^.

### Protocols of the simulation experiments

For the simulation protocol, we used the standard S1-S2 protocol to simulate tissue electrophysiology. In the S1-S2 protocol, the S2 stimulus was applied at the same position with varying coupling intervals after an action potential was activated by the last S1 stimulus. For the 2D ideal square tissue simulation, as shown in Figs 2, 6 different random seeds were used to calculate the average and standard deviation of VWs.

In the 2D tissue simulations, we used an isotropic domain size of 1000 × 1000 grid points for the 2D ideal tissue model, 600 × 600 grid points for the 2D circular ring tissue model and 672 × 644 grid points for the 2D ventricular model, which translated into physical sizes of 250 × 250 mm^2^, 210 × 210 mm^2^ and 235.2 × 225.4 mm^2^, respectively. All 2D tissue models were stimulated with a strength of −150 pA/pF and a stimulus duration of 3 ms. For the 3D model simulations, we used an isotropic domain size of 325 × 325 × 425 grid points for the 3D ventricular model, and all 3D tissue models were stimulated with a strength of −120 pA/pF and a stimulus duration of 2 ms. Furthermore, the stimulus area of the 2D ideal tissue was on the left side of the tissue, whose size was 3 × 0.25 × 1000 × 0.25 = 187.5 cm^2^. The stimulus area of the 2D circular ring and ventricular models were both in the intramyocardial area of the endocardium, whose size was 10 × 10 × 0.35 × 0.35 = 12.25 mm^2^. The stimulus area for the 3D ventricular models was also located in the intramyocardial area of the endocardium, and its size was less than 10 × 10 × 10 × 0.33 × 0.33 × 0.33 = 35.94 mm^3^.

## Supplementary information

Supplementary Information

## Data Availability

2D actual ventricular images are from Ventricular Infarct Segmentation challenge 2012^43^, http://www.cardiacatlas.org/challenges/ventricular-infarct-segmentation/.
