# Supplementary material for: Influence of the distribution of fibrosis within an area of myocardial infarction on wave propagation in ventricular tissue
Source: Sci Rep. 2019 Oct 2;9:14151. doi: 10.1038/s41598-019-50478-5 (PMC6775234; doi:10.1038/s41598-019-50478-5)
Supplement: Supplementary file 1 — Supplementary Information [file 41598_2019_50478_MOESM1_ESM.doc]

Influence of the distribution of fibrosis within an area of myocardial infarction on wave propagation in ventricular tissue

Cuiping Liang^1^, Kuanquan Wang^1*^, Qince Li^1*^, Jieyun Bai^1^, Henggui Zhang^1,2,3,4^

^1^ School of Computer Science and Technology, Harbin Institute of Technology, Harbin, Heilongjiang, China

^2^School of Physics and Astronomy, The University of Manchester, Manchester, UK

^3^Space Institute of Southern China, Shenzhen, China

^4^Key Laboratory of Medical Electrophysiology, Ministry of Education, Collaborative Innovation Center for Prevention and Treatment of Cardiovascular Disease/Institute of Cardiovascular Research, Southwest Medical University, Luzhou, China

^*^Correspondence and requests for materials should be addressed to W.K. and Q.L. ([wangkq@hit.edu.cn](mailto:wangkq@hit.edu.cn); qinceli@hit.edu.cn)

Supplement

**Simulations in single cell and 2D ideal tissues.** In single cell simulation, the action potential (AP) for the MI zone was characterized by decreased action potential amplitude (APA), a decreased maximum of depolarization rate (dV/dt_max) and increased action potential duration at 90% repolarization (APD_90_), as shown in Supplementary Figure S1 (a) & (b), similar to that reported previously[^1^](#_ENREF_1). Supplementary Figure S1 (c) shows wave propagation in 2D normal, infarcted tissue and infarcted tissue with different fibrosis at 100ms and 200ms.It demonstrated that conduction velocity decreased in infarcted tissue (47 cm/s) compared to that in normal myocardium tissue (67.25 cm/s in Supplementary Figure S1 (d)). In addition, in 2D MI fibrous tissue, conduction velocity further declines with an increase of fibrosis percentage. Wave conduct block occurred in 45% fibrous 2D tissue and conduction velocities are 44.50 cm/s, 38 cm/s, 27.25 cm/s and 24.25 cm/s respectively, in 5%, 15%, 25% and 35% ideal fibrosis tissue as shown in Supplementary Figure S1 (d).


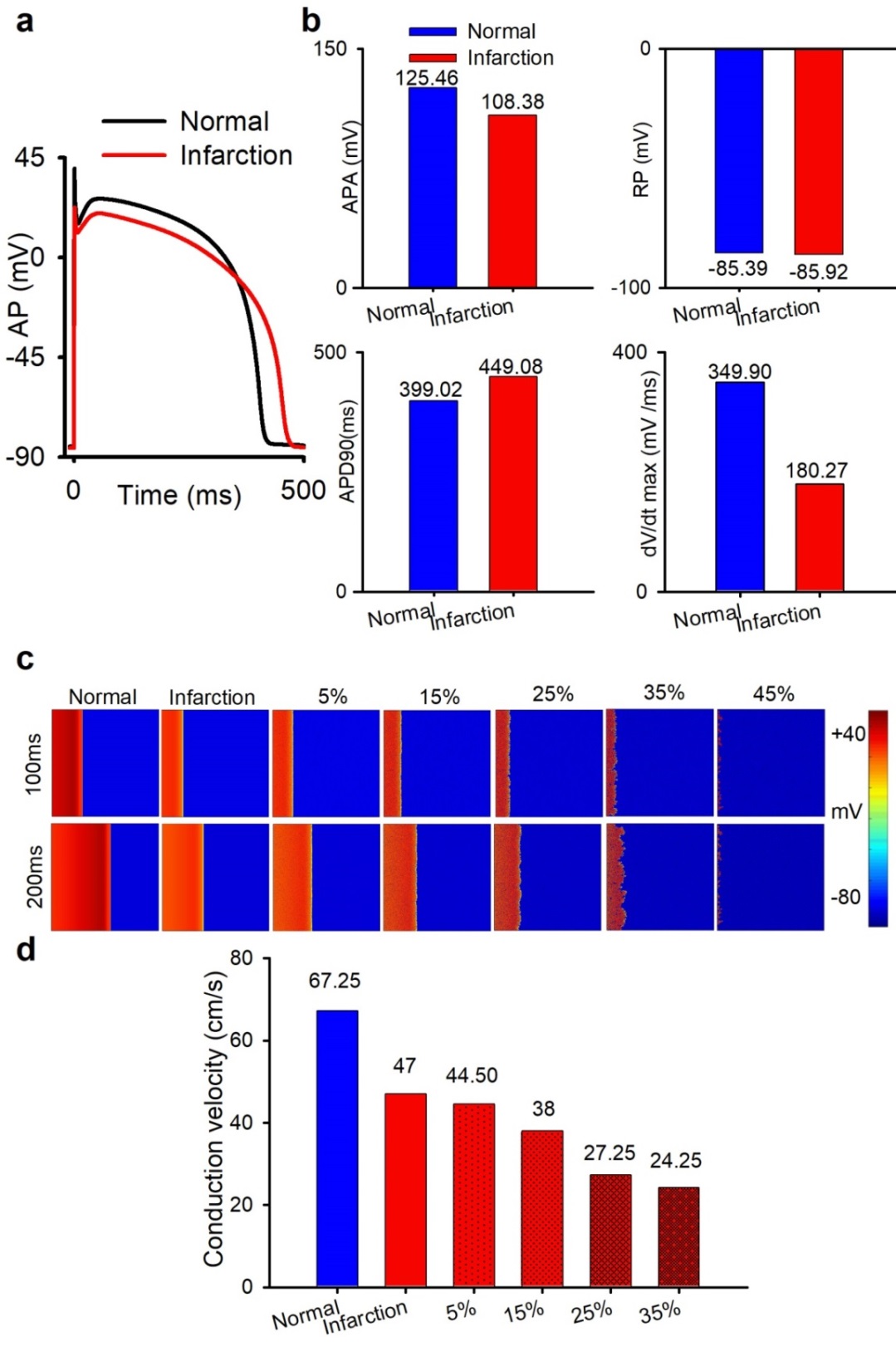


Supplementary Figure S1. Action potential characteristics of normal and infarcted midmyocardial myocytes as well as wave propagation and conduction velocity under normal, infarcted and infarction with fibrosis conditions. (a) and (b): Action potential (a) and the value of APA, RP, APD90 and d*V*/d*t* max (b) in normal and infarcted midmyocardial myocytes. (c) and (d): Wave propagation at 100ms and 200ms (c) and conduction velocity (d) in normal, infarcted, infarcted with 5%, 15%, 25%, 35% and 45% fibrosis in 2D ideal tissue.

**Gradient fibrosis distribution of 2D ideal square tissues.**

Supplementary Table S1. Gradient fibrosis distribution ranging from 6% to 31%.

| Number  of rings  Average  of fibrosis | 1 | 2 | 3 | 4 | 5 |
| --- | --- | --- | --- | --- | --- |
| 6% | 20% | 15% | 10% | 5% | 0% |
| 11% | 25% | 20% | 15% | 10% | 5% |
| 16% | 30% | 25% | 20% | 15% | 10% |
| 21% | 35% | 30% | 25% | 20% | 15% |
| 23% | 37% | 32% | 27% | 22% | 17% |
| 26% | 40% | 35% | 30% | 25% | 20% |
| 28% | 42% | 37% | 32% | 27% | 22% |
| 31% | 45% | 40% | 35% | 30% | 25% |

*Note: Number of rings from inside to outside is from 1 to 5.*

References

1 Deng, D., Arevalo, H. J., Prakosa, A., Callans, D. J. & Trayanova, N. A. A feasibility study of arrhythmia risk prediction in patients with myocardial infarction and preserved ejection fraction*. Europace : European pacing, arrhythmias, and cardiac electrophysiology : journal of the working groups on cardiac pacing, arrhythmias, and cardiac cellular electrophysiology of the European Society of Cardiolo*g**y** 18, iv60-iv66, doi:10.1093/europace/euw351 (2016).
